# Hospital Annual Meetings

**Published:** 1920-06-05

**Authors:** 


### Hospital Annual Meetings.

### Chelsea Hospital for Women.

The Chelsea Hospital for Women, the Annual Meeting of which has just been held, was able last year practically to wipe out the debt of £3,000 on the general fund, which accumulated in 1918. The Council, however, cannot but regard the future with anxiety in view of the ever-increasing expenditure and the urgent necessity for substantial increase in the regular income. Patients to the number of 760 were admitted to the wards in 1919, and no higher testimony, it is stated, can be given to the skill of the medical and nursing staffs than the fact that although the large majority of cases required major operations of a critical nature, the rate of mortality was a little below 1½ per cent. The number of patients treated at the convalescent home last year was 325, as compared with 265 in 1918. The office of consulting physician has been instituted at the hospital, because it occasionally happens that the surgeon in charge of a patient desires to consult a physician in regard to the case. Mr. J. A. Torrens, M.B., B.Sc., M.R.C.P., has been appointed to that position. The Council points out that the hospital may claim to be taking an active part in building up a new national life, inasmuch as the welfare of homes and children depends on the health of the mothers.

### Queen's Hospital for Children.

Prince Albert, the President, occupied the chair at the fifty-second Annual Meeting of Governors of the Queen's Hospital for Children. The work of the hospital has quite outgrown the size of the institution, and in the interests of the community it is urged that the proposed new buildings, which it is estimated will cost £50,000, should be provided without further delay. Expenditure had risen from £21,709 in 1918 to £27,181 last year, while income only reached £22,778, an increase, nevertheless, of over £2,500 as compared with the previous twelve months. The present year opened with a deficit of over £7,500. An appeal by means of a flag day was made last year in the neighbourhood of the hospital and surrounding district, but was abandoned, as far as the City and West End were concerned, in view, says the Committee, of the marked hostility shown to flag-sellers on nearly all occasions since the Armistice in those districts. A collecting-box scheme, however, was adopted instead, and proved more successful than the flag-selling could have been. These enterprises resulted in an amount of £1,573 being collected.

### South London Hospital for Women.

The Marchioness of Londonderry presided at the Annual Meeting of the South London Hospital for Women, Clapham Common, which was held at Londonderry House, Park Lane. Amongst those present were H.R.H. Princess Helena Victoria, who, in consequence of the bereavement in the Royal Family, did not address the meeting. The Marchioness of Londonderry gave a summary of the work carried out at the hospital during the past year, where 1,142 patients were treated. The out-patients had increased, but, unfortunately, because of the restricted accommodation, 4,613 women had to be turned away. Through the generosity of Sir Alfred Yarrow and Miss Eleanor Barnes a site was available for a new out-patient department, but the money to build it is not yet available. The urgent need for this new department is shown by the fact that the attendances of out-patients have risen from 14,000 in 1913 to over 33,000 last year. Unfortunately, another difficulty the Committee have to face is a lack of sufficient staff, and through this cause the children's ward had to be closed five months during last year. The expenditure was £1,396 more than in the previous year.

### Victoria Hospital for Children.

The Victoria Hospital for Children, Chelsea, the Annual Meeting of which was held recently, has remedied the staff shortage from which it has been suffering, and the whole of the accommodation at the institution is in consequence once more available. In-patients admitted during 1919 numbered 1,041, while the number of new out-patients in that period was 13,137, as compared with 12,228 a year previously. The financial position is causing the Committee the gravest anxiety, the total income received last year amounting to £10,685, as against £13,551 in 1918—a decrease of £2,886. Expenditure, on the other hand, increased from £11,078 in 1918 to £14,349 during the period under review. To provide funds to meet that expenditure the Committee has been obliged to sell at great loss some of the hospital investments, and more will have to be disposed of unless income materially increases. The damage done to the Convalescent Home at Broadstairs during the air-raids has been repaired, and the Home is now in use. With a view to extending it so as to provide separate accommodation for children suffering from heart complaints, chorea, and other ailments requiring complete rest and quiet, the adjoining property, "Mascotte," has been acquired. The Home at Biggin Hill is still in the possession of the Military authorities.

### Hospital for Sick Children.

Lord Aberdare occupied the chair at the Annual Meeting of Governors of the Hospital for Sick Children, Great Ormond Street, held recently. In-patients admitted during 1919 numbered 2,727, being an increase of 160 as compared with the previous year, while new out-patients totalled 30,843—3,944 more than in 1918. Out-patient attendances last year exceeded 100,000. The heavy diminution of £11,351 in the total income of the hospital took place in 1919 as compared with the previous twelve months, while total ordinary expenditure increased by £6,653. The year under review closed with a debt to the bankers of £6,500. The Committee desires to bring to the notice of the public the proposal to establish an auxiliary hospital in the country in connection with the parent hospital in Great Ormond Street. The fact that the institution has no convalescent home under its direct supervision is the present reason for its establishment. Such an institution would, it is stated, mark a step forward in the treatment of sick children. The Committee is convinced that it is one of national importance, which should meet with wide public recognition and support at a time when the importance of child-welfare has become one of the first problems of the State.

